# Characterization of a Bvg-regulated fatty acid methyl-transferase in *Bordetella pertussis*

**DOI:** 10.1371/journal.pone.0176396

**Published:** 2017-05-11

**Authors:** Alex Rivera-Millot, Elodie Lesne, Luis Solans, Loic Coutte, Justine Bertrand-Michel, Philippe Froguel, Véronique Dhennin, David Hot, Camille Locht, Rudy Antoine, Françoise Jacob-Dubuisson

**Affiliations:** 1 Univ. Lille, CNRS, Inserm, CHU Lille, Institut Pasteur de Lille, U1019- UMR 8204-CIIL-Centre d’Infection et d’Immunité de Lille, Lille, France; 2 MetaboHUB-MetaToul-Lipidomic Core Facility, Inserm U1048, Toulouse, France; 3 University of Lille, CNRS, Institut Pasteur de Lille, UMR 8199, European Genomic Institute for Diabetes, Lille, France; 4 Department of Genomics of Common Diseases, School of Public Health, Imperial College London, Hammersmith Hospital, London, United Kingdom; Universidad Nacional de la Plata, ARGENTINA

## Abstract

The whooping cough agent *Bordetella pertussis* controls the expression of its large virulence regulon in a coordinated manner through the two-component signal transduction system BvgAS. In addition to the genes coding for *bona fide* virulence factors, the Bvg regulon comprises genes of unknown function. In this work, we characterized a new Bvg-activated gene called *BP2936*. Homologs of *BP2936* are found in other pathogenic *Bordetellae* and in several other species, including plant pathogens and environmental bacteria. We showed that the gene product of *BP2936* is a membrane-associated methyl-transferase of free fatty acids. We thus propose to name it FmtB, for fatty acid methyl-transferase of *B**ordetella*. The role of this protein was tested in cellular and animal models of infection, but the loss of *BP2936* did not appear to affect host-pathogen interactions in those assays. The high level of conservation of *BP2936* among *B*. *pertussis* isolates nevertheless argues that it probably plays a role in the life cycle of this pathogen.

## Introduction

*Bordetella pertussis* is the agent responsible for whooping cough, an acute respiratory disease that affects exclusively humans [1]. This bacterium infects mainly naive individuals, in particular infants, and it is responsible for more than 300,000 deaths each year [2]. Whooping cough also affects adults and adolescents, in whom it causes milder forms of illness [3]. The high morbidity rate associated with this infection is explained by the aerosol mode of transmission of the bacterium, and by the limited duration of the immunity provided by vaccines [4].

*B*. *pertussis* produces an array of virulence factors, the expression of which is regulated by a two-component system called BvgAS [5]. BvgS and BvgA are the sensor-kinase and the response regulator of this system, respectively [6]. At 37°C, BvgS autophosphorylates and then transfers its phosphoryl group to BvgA via a complex phospho-transfer cascade [7]. Phosphorylated BvgA serves as a transcriptional activator of a number of genes and operons, including the *bvgAS* operon itself, that are called the virulence-activated genes (*vags*) [8]. In the conditions under which the *vags* are expressed, *B*. *pertussis* is in the virulent, Bvg^+^ phase. Major pathogenicity factors regulated by BvgAS include adhesins, such as fimbriae, the filamentous hemagglutinin FHA and several autotransporters, and toxins, including adenylate cyclase/hemolysin, dermonecrotic toxin and pertussis toxin [9]. *B*. *pertussis* is also a master at manipulating host immunity to its own advantage, with most virulence factors modulating the immune response in various ways [10].

BvgS works as a kinase by default. Signals that make BvgS switch to a phosphatase mode of activity in laboratory conditions include low temperatures and chemical modulators such as nicotinic acid and sulfate ions at millimolar concentrations [11,12]. In the so-called Bvg^-^ phase the *vags* are silent, while the so-called virulence-repressed genes (*vrgs*) are expressed at high levels. In the Bvg^-^ phase of *Bordetella bronchiseptica*, a closely related species that infects various mammals and can survive in the environment, a number of *vrg*s, notably coding for proteins involved in nutrient acquisition and motility functions, are expressed [12,13]. Many of those genes have been lost or are inactive in *B*. *pertussis*, which has undergone massive genome decay [14,15]. An intermediate ‘Bvg^i^’ phase has been identified that is triggered by intermediate concentrations of modulators [16]. In those conditions, adhesin genes are expressed but not those coding for toxins, leading to the hypothesis that this phase might be involved in the first stage of colonization or in the transmission of the bacterium between hosts.

The chemical modulators have widely been used as tools to study the Bvg regulon. Transcriptomic analyses have thus compared the Bvg^+^, Bvg^-^ and Bvg^i^ phases [17–19]. While they largely confirmed the Bvg regulation of the *bona fide* virulence genes discovered in the pre-genomic era, they also revealed that some of them are subject to additional regulation [19]. In addition, they unveiled new Bvg-regulated genes that have no assigned function. *BP2936* (numbering of the *B*. *pertussis Tohama* I reference genome) is one such gene. Orthologs of *BP2936* are found in other *Bordetella* species and other bacterial genera, including bacterial pathogens, but their function is unknown. In this work, we performed the first characterization of *BP2936*, a new *vag* member of the Bvg regulon.

## Materials and methods

### Strains and plasmids

BPSM, a *Tohama* I streptomycin- resistant derivative, and its derivatives BPRA (BPSM deleted of the *ptx* operon) and BPGR4 (BPSM deleted of the *fhaB* gene) were described earlier [20–22]. Modified Stainer Scholte liquid medium and Bordet Gengou blood-agar medium were used with the appropriate antibiotics to culture the *B*. *pertussis* strains, except for the lipid analyses (see below). To construct a *BP2936* deletion in BPSM, sequences flanking the gene on each side were amplified by PCR using the pairs of oligonucleotides 5'-gaattccgtgcaggtcgaagccaacaacga-3' and 5’-ggatcccgcgcgatgggagatgagag-3’, and 5'-ggatccgcctgacgctcgccggta-3' and 5’-aagcttcgcaaaggccgtgacatgggaca-3'. For all the PCR performed in this work, the amplicons were first introduced into pCRII-TOPO (Invitrogen) and sequenced. The two amplicons were introduced as EcoRI-BamHI and BamHI-HindIII fragments into pUC18 by ligation, after which the EcoRI-HindIII insert of the resulting plasmid was introduced into the mobilizable plasmid pSS1129 [23]. Allelic replacement in BPSM was performed by conjugation as described in [24], resulting in BPRM1. The deletion of *BP2936* in BPRM1 was verified by PCR and immunoblotting.

To complement the deletion, the *BP2936* gene, including the promoter region, was amplified by PCR using 5'-ggtacccgacttcaaccgcggcgcatt-3' and 5'-ggatccaaagggcggaactaccggaca-3' as primers. In addition, internal sequences of the *ureJ* and *ureC* genes were amplified, using 5'-gaattcgccgccctgatgctgttctcg-3' and 5'-ggtaccgacacctccctggtcagaccc-3', and 5'-ggatccatgaccaggatttcgcgttcgg-3' and 5'-aagcttccagcgcctgcagcatgg-3' as primers, respectively. After sequencing, the 3 amplicons were successively introduced into pCU18 by ligation, in such a way as to flank *BP2936* on both sides with the *ure* fragments. The rest of the procedure was as described above, using BPRM1 as the recipient for conjugation. This yielded BPRM2, which harbors *BP2936* in the *ure* locus. The *BP2936*-containing amplicon was also introduced as a KpnI-BamHI fragment in pBBR1-MCS5, a replicative, low-copy plasmid [25], resulting in pBP2936. This plasmid was used for complementation of BPRM1.

Point mutations were introduced in *BP2936* by site-directed mutagenesis with the Quik Change II XL kit (Agilent). pUC18 containing the *BP2936* gene was used as the template. After sequencing, the mutated gene was introduced in pBBR1-MCS5, yielding pBP2936^DA/YS^.

Several constructs were generated for the production of the protein encoded by *BP2936* in *Escherichia coli*. Initially, the oligonucleotides 5’-ggatccgacgctggcgtggccatctg-3’ and 5’-aagcttcaggccggcttgcgggcgat-3’ (called rec-Hind) were used to amplify the longer putative cds. The resulting amplicon was restricted by BamHI and HindIII and ligated with pQE30 (Qiagen). This introduced a 6-His tag at the N-terminus of the protein. A shorter PCR was performed in a similar manner, using 5’-ggatcccatcgtgcagcgtcggccgat-3’ and rec-Hind as primers, and the amplicon was introduced in the same vector. *BP2936* carrying the two point mutations was also amplified and cloned similarly.

### RNA sequencing

RNA-seq was performed with two independent cultures stopped in the exponential phase of growth (OD_600 nm_ = 2) by adding 2 ml of 5:95 (v:v) phenol/ethanol to 8 ml of bacterial suspension. Bacteria were pelleted by centrifugation, and total RNA was extracted using TriReagent (Invitrogen). Genomic DNA was removed by two steps of DNase I treatment (Sigma Aldrich). Total RNA was treated with the Ribo-Zero rRNA Removal Kits (Illumina) following the manufacturer’s recommendations. The rRNA-depleted RNA were then used to prepare the two Illumina libraries using the TruSeq RNA Library Preparation Kit (Illumina), following sequencing on an Illumina NextSeq 500 benchtop sequencer on SR150 high output run mode.

Differential RNA-seq was performed according to the protocol described in [26]. Briefly, three cultures were stopped either in the exponential (OD_600 nm_ ~ 1.5), late exponential (OD_600 nm_ ~ 2.4) or early stationary phases (OD_600 nm_ ~ 3.2) by adding 2 ml of 5:95 (v:v) phenol/ethanol to 8 ml of bacterial suspension. Bacteria were pelleted by centrifugation, and total RNA was extracted using TriReagent (Invitrogen). Genomic DNA was removed by two steps of DNase I treatment (Sigma Aldrich) before pooling. To digest non-primary transcripts, 5 μg of total RNA were then treated with 1U of Terminator^TM^ 5’phosphate-dependant exonuclease (TEX) (Epicentre) for 60 min at 30°C. The reaction was stopped by adding 1 μl of 100 mM EDTA pH 8, and the RNA were purified by an organic extraction. TEX-treated RNA were incubated with 1U of Tobacco Acid Pyrophosphatase (TAP) (Epicentre) for 60 min at 37°C before organic extraction. Sequencing libraries of cDNA were constructed with 500 ng of TEX/TAP-treated RNA using the Ion Total RNA-seq Kit v2 (Life Technologies), following the manufacturer’s recommendations. Libraries were purified twice with 1.8 volume of Agencourt AMPure (Beckman Coulter). Emulsion PCR, enrichment and sequencing were made using the Ion PGM^TM^ template OT2 400 Kit and the Ion PGM^TM^ sequencing 400 Kit. Enriched beads were sequenced on an Ion Torrent PGM machine using a 318 v2 chip. RNA-seq and dRNA-Seq sequencing reads results were then imported and mapped on the *B*. *pertussis* TohamaI genome using the CLC Genomics Workbench software (QIAGEN Bioinformatics).

### Protein techniques

The recombinant pQE30 plasmids were introduced in *E*. *coli* M15(pREp4) for protein expression. The cells were cultured in LB medium, and induction of the recombinant proteins was performed with 1 mM IPTG for 3 hours. The bacteria were resuspended in 20 mM Tris-HCl (pH 7.5), 100 mM NaCl, 10 mM imidazole (TNI buffer) and broken by passages in a French pressure cell. The lysates were clarified by centrifugation. The longer protein, which was used for antibody production, formed inclusion bodies, and thus the insoluble pellets were resuspended in 50 mM Tris-HCl (pH 7.5), 150 mM NaCl, and 6 M urea and rocked gently for one hour. The solution was clarified by centrifugation and the protein was purified by chromatography on a Ni^++^ column. Polyclonal antibodies were raised in rats (Eurogentec, Belgium). The shorter protein was soluble and purified by Ni^++^ chromatography in TNI buffer. It was concentrated by ultrafiltration to 2 mg/mL for the enzymatic assays.

The fractionation of *B*. *pertussis* for detection of the protein coded by *BP2936* was performed as follows. The bacteria were grown in SS medium until exponential phase, collected by centrifugation, resuspended in 50 mM Tris-HCl (pH 8) with a tablet of Protease inhibitor (Complete, Roche) and lysed in French pressure cell. The clarified lysate was ultracentrifuged at 100,000 g for one hour to separate the soluble and membrane fractions. The samples were analyzed by immunoblotting using the antibodies raised against the longer recombinant protein. The loading of the samples was standardized based on the optical densities of the cultures when the bacteria were collected for processing. The immunoblots were developed by chemiluminescence (ECL Prime, Amersham).

### Homology modeling

A three-dimensional model of the protein was constructed with S-adenosyl-L-homocysteine (SAH) and palmitic acid in its enzymatic cavity. Briefly, the crystal structures of the full *B*. *parapertussis* ortholog [Protein Data Bank (PDB) entry 3OCJ] and of the SAH-binding domain of a methyl-transferase from *Pyrococcus horikoshii* OT3 (PDB entry 1WZN) were used as templates in MODELLER version 9.17 [27]. Templates were structurally aligned using PROMALS3D [28]. Molecular structure inspections and illustrations were made using PyMOL (PyMOL Molecular Graphics System, version 1.8.2, Schrödinger).

### *In vitro* assay of methyl-transferase activity

A kit developed by Caiman Chemicals was used for measurements of methyl-transferase activity. Briefly, the kit detects a product of the methyl-transferase reaction, SAH, by a series of enzymatic reactions that eventually produce resorufin, a fluorescent molecule. The shorter, soluble recombinant protein was used in this assay, with palmitate as the substrate at concentrations ranging from 0.4 mM to 20 mM. Palmitate was either solubilized in 2% Triton X100 or presented to the enzyme as a BSA conjugate, which was prepared according to the instructions of the manufacturer of fatty-acid-free BSA (Seahorse Bioscience). The assays were conducted in 96-wells plates. The excitation wavelength was 535 nm and the detection wavelength 585 nm.

### Quantification of free methylated fatty acids in *B*. *pertussis*

BPSM and its derivatives were cultured in Thijs medium, which is devoid of casaminoacids, to prevent contaminating the samples with lipids from the medium [29]. The culture flasks were disposable, and the glassware used for the rest of the procedure was first washed with a mix of chloroform and methanol to remove traces of lipids. The bacteria were collected by centrifugation at the end of the exponential phase, washed twice in sterile PBS and diluted to 5x10^10^ bacteria per tube. They were killed by the addition of 1 mL chloroform. Lipids were extracted as described in [30] after addition of dichloromethane (1.5 ml), methanol (2.5 ml) and water (2.1 ml) in the presence of an internal standard, methylated heptadecanoate acid (4 μg). The lipid extract was then evaporated to dryness and dissolved in ethyl acetate (20 μl). Methylated fatty acids were analyzed on a ThermoScientific Trace GC coupled to a Trace ISQ Mass selective detector (ThermoScientific). The fatty acids were separated on an Agilent J&W HP-5MS capillary column (30 m, 0.25 mm, 0.25 μm phase thickness). The oven temperature program was as follows: 180°C for 1 min, 20°C/min to 250°C, 5°C/min to 300°C where the temperature was kept for 8 min, and then 35°C/min to 325°C. High-purity helium was used as carrier gas at a flow rate of 0.8 mL/min in constant flow mode. The samples were injected in a splitless mode with an injection volume of 1 μL. The injector, transfer line and source temperatures were 270°C, 280°C and 250°C, respectively. The mass spectrometer was operated in the full scan mode. Peak detection, integration and relative quantification analysis were executed using Xcalibur Quantitative browser (ThermoScientific).

### Infection of macrophages

The monocytic human cell line THP1 was used in these experiments. The cells were cultured in RPMI medium with 10% fetal calf serum and 0.05 mM β mercaptoethanol. They were treated with 20 ng/mL phorbol 12-myristate 13-acetate (Sigma) for 18 h to induce their differentiation into macrophages. The bacteria were grown at 37°C on BG blood-agar plates, scraped from the plates after 48 hours and resuspended in PBS. They were used at a multiplicity of infection of 200. The bacteria were centrifuged onto the cells at 640 g for 5 min, and incubation was performed for 30 min at 37°C with 5% CO_2_. After a wash in PBS, polymyxin B was added to a concentration of 100 μg/mL to eliminate extracellular bacteria. After 1 h the concentration of polymyxin B was lowered 20 fold. Cells were washed in PBS and then lysed with 0.1% saponine 1 h, 4 h and 24 h after infection. Serial dilutions were plated on BG blood agar to count the bacteria. The experiments were performed in triplicates.

### Animal experiments

The bacterial strains used for the animal experiments were grown on plates for 48 h, scraped and resuspended in sterile PBS to 10^6^ bacteria per 20 mL. Female, 6-weeks-old JAX ™ BALB/cByJ mice from Charles River were anesthetized intraperitoneally with a mixture of ketamine, atropine and valium and infected by intranasal inoculation with 10^6^ viable bacteria (S1 Fig). Groups of 3 or 4 animals per bacterial strain were sacrificed after 3 h, and 4, 7, 14 or 21 days post-inoculation in the first experiment, and after 3 h and 5 days post-inoculation in the second. Their lungs were removed in a sterile manner and homogenized using an Ultra Thurax apparatus. Serial dilutions were performed in PBS and plated to count the bacteria. All the experiments were carried out in accordance with the guidelines of the French Ministry of Research regarding animal experiments, and the protocols were approved by the Ethical Committees of the Region Nord Pas de Calais and the Ministry of Research (agreement number APAFIS#9107–201603311654342 V3).

## Results

### Genomic context of *BP2936*, a new virulence-activated gene

Transcriptomic analyses comparing the Bvg^+^ and Bvg^-^ phases of *B*. *pertussis* have unveiled uncharacterized genes that are regulated in a similar manner as the *bona fide* virulence genes [17]. One of these genes, numbered *BP2936* in the genome of the reference strain *Tohama* I, has been annotated as coding for a putative exported protein of unknown function [14]. In addition to being positively regulated by BvgAS, *BP2936* is part of a subset of *vags* whose regulation is also under the control of another transcriptional activator called RisA [19].

**Fig 1:**
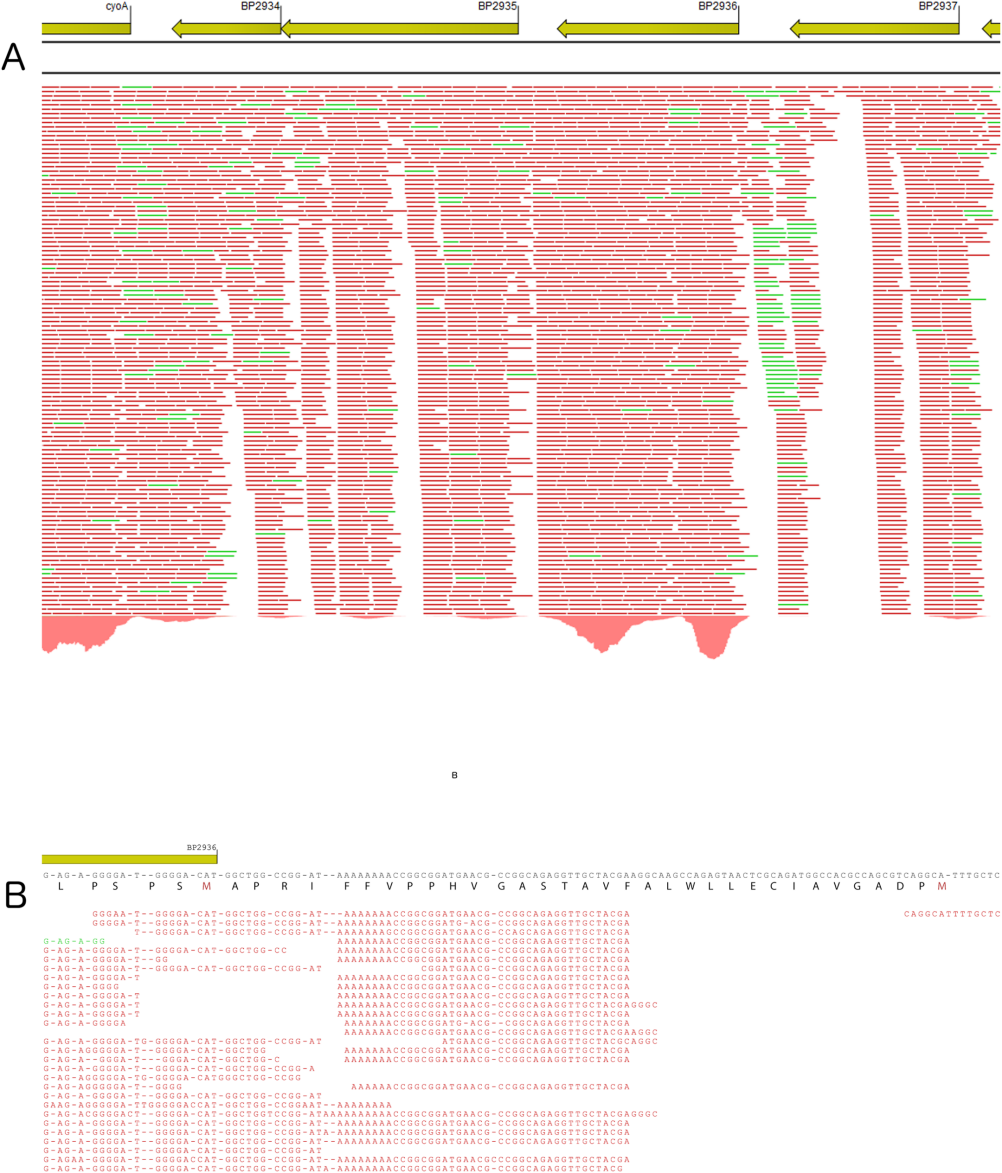
Characterization of the *BP2936* locus by RNA seq. A. Representation of the deep RNA sequencing results for the locus encompassing *BP2936*. *BP2936* is encoded by the anti-sens strand of the genome. The red and green bars under the genes represent transcripts of the anti-sense and sense strands, respectively. B. Differential RNA sequencing was used to identify the transcription initiation site of BP2936. The consensus sequence is indicated. Translation of the coding DNA sequence is shown from right to left, indicating the two possible initiation Met. The primary transcripts identified by dRNA seq show that the transcription start site maps between these two codons, at nucleotide position 3125512 in the genome of *Tohama* I.

Deep RNA sequencing analyses (RNA-seq; Fig 1A) indicated that *BP2936* forms a monocistronic operon. It is flanked on one side by an operon (*BP2935-BP2934*) coding for a two-component system called RegAB and potentially involved in the redox response, itself adjacent to cytochrome ubiquinol oxidase genes. On the other side of *BP2936* is found another gene of unknown function, *BP2937*. The flanking genes are coded on the same DNA strand, but RNA-seq analyses indicated that they are not co-transcribed with *BP2936*.

*BP2936* has two potential initiation codons, which would yield a protein of 302 or 335 residues. The current annotation of the *Tohama* I genome predicts that the coding DNA sequence (cds) starts at the first of the two putative initiation codons. A hydrophobic segment that does not have the features of a signal-peptide is thus found in the N-terminal region of the predicted cds (Fig 1B).

BLAST analyses of the predicted protein sequence indicated that the product of *BP2936* is most likely a putative S-adenosyl-methionine (SAM)-dependent methyl-transferase. The closest homologues are found in other pathogenic *Bordetellae* (see below), in various Gram-negative bacteria, including human or plants pathogens such as *Legionella pneumophila*, *Burkholderia cepacia*, *Dickeya dadantii*, *Ralstonia solanacearum*, *Pectobacterium carotovorum* or *Xanthomonas campestris*, and in several environmental species. No characterization of these proteins has been reported.

### *BP2936* in pathogenic *Bordetellae*

There are currently 450 genomic sequences of isolates in the *bronchiseptica* complex, which encompasses the three closely related species *B*. *pertussis*, *Bordetella parapertussis* and *Bordetella bronchiseptica* [31]. We performed *in silico* analyses of these sequences, which showed that *BP2936* is present in all genomes except for that of *B*. *pertussis* 18323. In this isolate, a 31-kbp deletion between two *IS481* copies encompasses 34 cds, including *BP2936*. The sequence and the genomic context of *BP2936* are both extremely well conserved. Among the available *B*. *pertussis* genomic sequences, we found no case of an insertion sequence in *BP2936*, suggesting that this gene is not subject to the global genomic reduction that characterizes *B*. *pertussis* [31,32]. Orthologs of *BP2936* are found in the related species *Bordetella holmesii*, *Bordetella hinzii*, *Bordetella pseudohinzii*, *Bordetella avium*, *Bordetella trematum*, but not in the environmental species *Bordetella petrii*. Within the *bronchiseptica* complex the predicted proteins are identical to 97%. The putative proteins of the other *Bordetella* species are approximately 50% identical to the product of *BP2936*. The genetic organizations of the locus in the other species are different from that in the *bronchiseptica* complex.

### Detection of the *BP2936* gene product

A deletion of *BP2936* was generated in the *Tohama* I derivative BPSM by allelic exchange, yielding BPRM1. To complement this deletion, the *BP2936* gene was introduced by homologous recombination into the *ure* locus, which is inactive in *B*. *pertussis*. This yielded BPRM2. BPRM1 was also complemented by the introduction of *BP2936* under the control of its own promoter on a replicative plasmid, called pBP2936.

**Fig 2:**
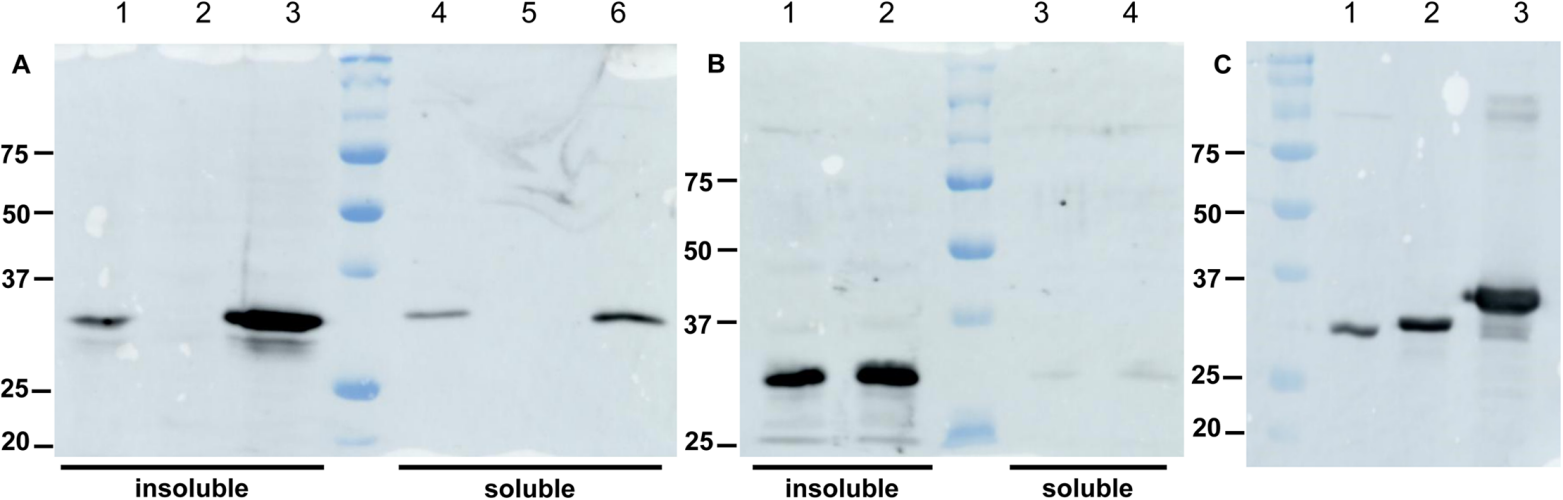
Detection of the product of *BP2936* by immunoblot analyses. (A), insoluble and soluble fractions of BPSM (wt control; lanes 1 and 4), BPRM1 (deletion mutant; lanes 2 and 5) and BPRM1(pBP2936) (mutant complemented on plasmid; lanes 3 and 6) were analyzed by immunoblotting with antibodies raised against the recombinant protein. (B), the same experiment was performed with the insoluble and soluble fractions of BPSM (lanes 1 and 3) and BPRM2 (mutant complemented on chromosome; lanes 2 and 4). (C), a comparison of the sizes of the three proteins, i.e., the 2936P protein produced by *B*. *pertussis* (lane 1), and the recombinant proteins rec2936P-sh (lane 2) and rec2936P-lg (lane 3) produced in *E*. *coli*, was performed. Note that 2936P migrates slightly faster than the shorter recombinant protein, most likely because of the purification tag added to the latter.

A recombinant protein was produced in *Escherichia coli* to raise polyclonal antibodies. The first potential initiation codon was used, and a 6-His tag was added at the N-terminus of the recombinant protein to facilitate purification. The recombinant protein called rec2936P-lg formed inclusion bodies, and it was thus purified in the presence of urea. Antibodies were raised against rec2936P-lg. Immunoblot analyses showed that they recognized a protein from *B*. *pertussis* migrating at approximately 30 kDa in BPSM, but not in BPRM1. This demonstrated that the *BP2936* gene is expressed in *B*. *pertussis* grown under usual laboratory growth conditions. The chromosomal complementation of the deletion restored the production of the protein to wild type (wt) levels in BPRM2, and to higher levels in BPRM1(pBP2936), in which the gene is carried by a plasmid (Fig 2A and 2B).

The *B*. *pertussis* protein, called 2936P, was found to migrate faster than the recombinant rec2936P-lg protein produced in *E*. *coli* (Fig 2C), indicating that the second putative initiation codon is used in *B*. *pertussis*. This is consistent with the mapping by differential RNA sequencing (dRNA seq) of the transcription initiation site, which was found to be located at nucleotide position 3125512 in the genome of *Tohama* I, between the two putative initiation codons (Fig 1B). Therefore, the protein encoded by *BP2936* is 302-residue long and devoid of a hydrophobic segment or signal sequence. It should thus be cytoplasmic. Intriguingly, however, fractionation of BPSM cell extracts between soluble and membrane fractions showed that most of 2936P was found in the latter (Fig 2A and 2B). This concurs with proteomic data that identified 2936P in membrane-enriched fractions of *B*. *pertussis* [33].

### Activity of the protein coded by *BP2936*

**Fig 3:**
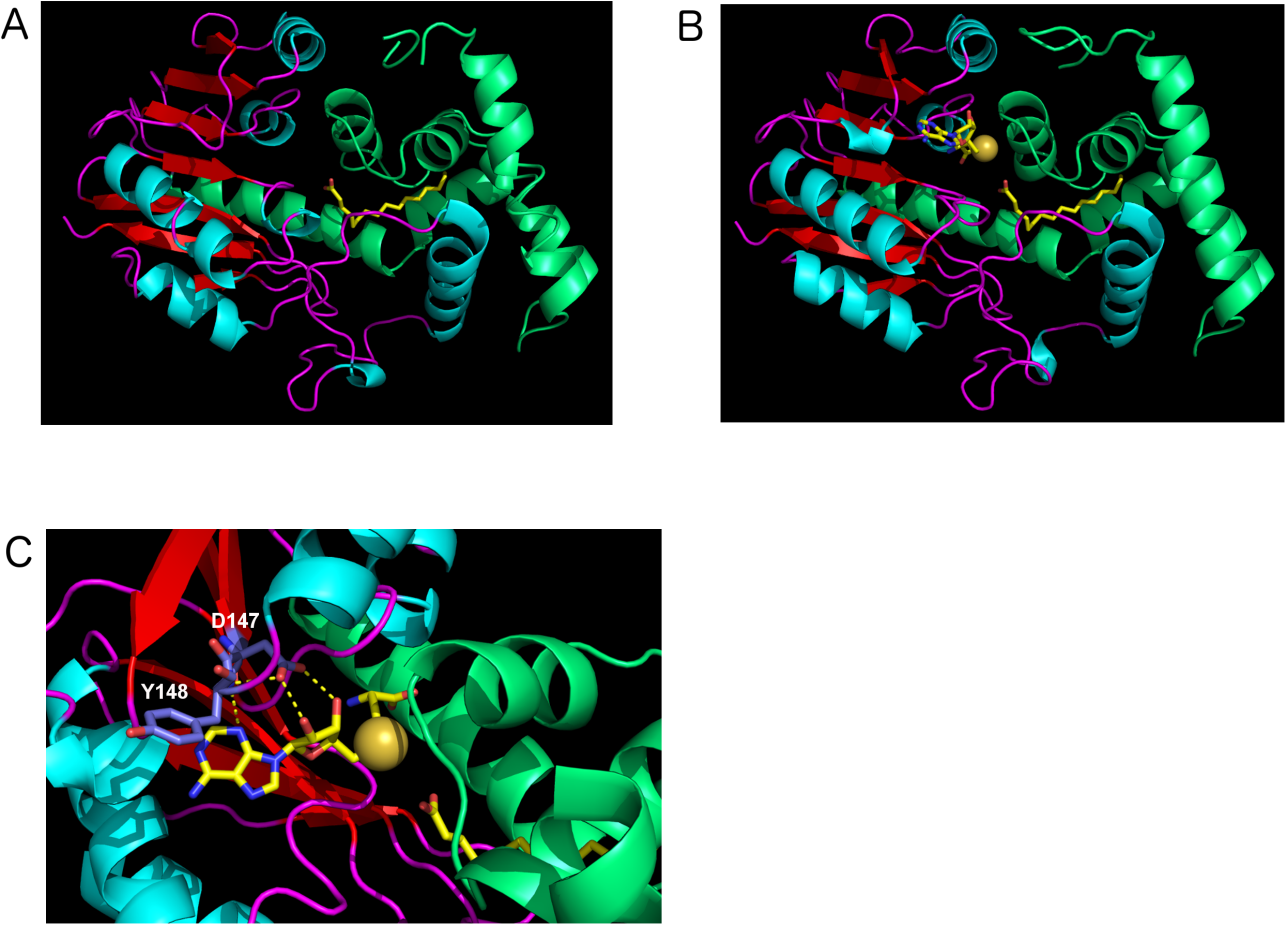
Structure of the protein coded by the *B*. *parapertussis* ortholog of *BP2936*. The coordinates found in the RCSB Protein Data Bank (Nr 3OJC) correspond to the *B*. *parapertussis* ortholog (97% sequence identity with 2936P). (A), the structure of the protein coded by BPP1064 is shown in cartoon representation. The palmitate molecule found in the cavity is shown in yellow stick representation. (B), A model of 2936P with palmitate and S-adenosyl homocysteine (SAH) in the cavity was constructed. Both SAH and the methyl palmitate are displayed in sticks. A zoom of the relevant region is presented in C. The sulfur atom of SAH, which carries the methyl group in SAM, is shown as a yellow ball. The conserved residues modified by site-directed mutagenesis (colored in slate blue) are indicated.

SAM-dependent methyl-transferases are a large class of enzymes whose possible substrates include DNA, RNA, small molecules or proteins [34]. Interestingly, the X-ray structure of the protein coded by *BPP1064*, the *BP2936* ortholog of *B*. *parapertussis*, has been reported by another group thanks to a program of structural genomics (Protein Data Bank number: 3OJC). As the sequences of the two proteins are > 97% identical, we used the structure of the *B*. *parapertussis* protein as a model for 2936P. The putative SAM-binding domain of the *B*. *parapertussis* ortholog adopts a typical Rossman-like fold, widespread among methyl-transferases [34]. Basically, it is composed of alternating α helices and β strands, with the latter forming a central β sheet flanked by α helical layers on both sides (Fig 3A). The functionally important SAM-binding residues that are conserved among SAM-dependent methyl-transferases and located at the C termini of the β strands were found in the *B*. *parapertussis* ortholog of 2936P [34–38]. The less conserved substrate-binding domain, which is all β helical in this case, crystallized with a small molecule, most likely a palmitic acid, in its cavity (Fig 3A).

The X-ray structure of another SAM-dependent methyl transferase that co-crystallized with S-adenosyl homocysteine (SAH), the reaction product of SAM, was used for structural alignments with that of the *B*. *parapertussis* enzyme. We were thus able to predict the position of SAH in the cavity of the protein, revealing that the carboxylate group of the palmitate molecule might be oriented in an appropriate manner for methylation in a SAM-dependent manner (Fig 3B and 3C). The reaction product would thus be a methyl palmitate, suggesting that the protein coded by *BP2936* may be a fatty acid methyl-transferase.

In order to test this hypothesis, a new recombinant protein corresponding to the shorter ORF was prepared with an N-terminal His tag. This protein, called rec2936P-sh, was produced in *E*. *coli* and purified in a soluble form (Fig 2C). Structural alignments of SAM-dependent methyl-transferases have identified a conserved motif Ile-(Asp/Glu)-Tyr in the second β strand. The acidic residue located at the edge of the β sheet is involved in the binding of SAM, and the aromatic residue might also form pi interactions with its adenine moiety [34–39] (Fig 3C). To disrupt the putative enzymatic activity of rec2936P-sh, the Asp^147^ residue that corresponds to the acidic residue of the conserved SAM-binding motif was replaced by Ala. Similarly, the following Tyr^148^ residue that may also contribute to SAM binding was replaced with a Ser. These two substitutions were combined in a new recombinant protein, called rec2936P-sh^DA/YS^. It was produced in *E*. *coli* and purified like its wt counterpart.

The two recombinant proteins were used in an *in vitro* assay of methyl-transferase activity. This assay detects the production of SAH by a series of successive reactions that yield a fluorescent product, resorufin. However, the background reaction in the absence of enzyme proved to be very high in our hands, and no clear increase of activity was detected in the presence of rec2936P-sh (not shown). In an attempt to enhance substrate accessibility, we also conjugated palmitate to fatty-acid free BSA, to optimize its presentation to rec2936P-sh. This did not reveal enzymatic activity either.

We thus turned to the detection of free methylated fatty acids produced by *B*. *pertussis*. We introduced the Asp to Ala and Tyr to Ser substitutions in the complementation plasmid, yielding pBP2936^DA/YS^. The level of production of the variant protein from this plasmid in *B*. *pertussis* was similar to that of the wt protein produced from the parent plasmid. Total *B*. *pertussis* cell extracts of BPSM, BPRM1, BPRM2, BPRM1(pBP2936) and BPRM1(pBP2936^DA/YS^) were prepared for the detection of methyl-ester fatty acids by mass spectrometry analyses. The classical protocol to quantify cellular fatty acids involves an acid methanolysis step, which was omitted in order to detect exclusively the naturally occurring fatty acids methylated on their carboxylate group.

**Fig 4:**
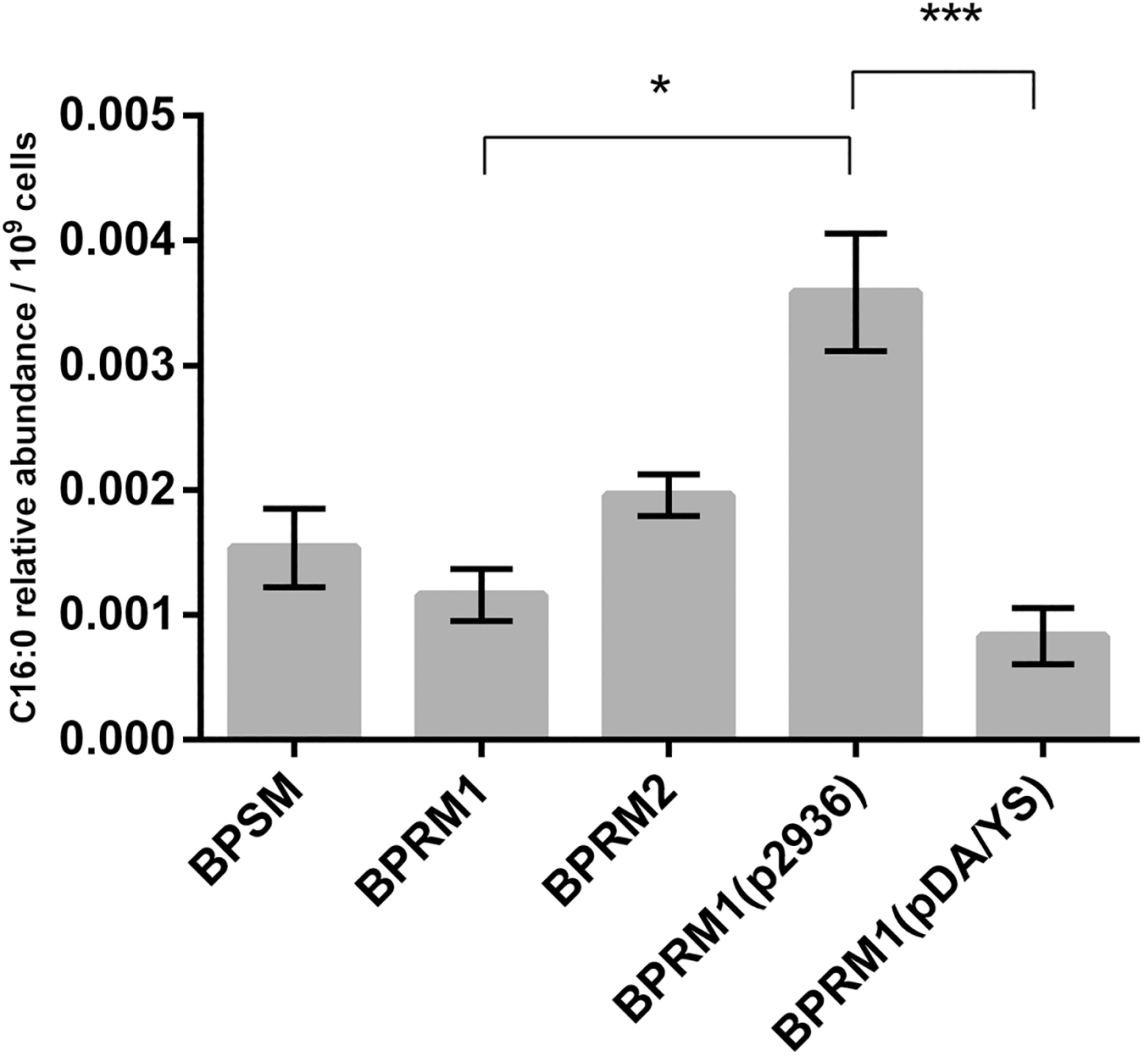
Detection of methyl fatty acids in *B*. *pertussis*. The amounts of methyl fatty acids were determined in BPSM (wt control), BPRM1 (deletion mutant), BPRM2 (chromosomal complementation), BPRM1(pBP2936) (plasmid complementation) and BPRM1(pBP2936^DA/YS^) (complementation on plasmid with mutated *BP2936*). The relative abundance is defined as the ratio between the area of the C16 peak and the area of the internal standard peak, normalized to the number of cells. The experiments were performed on 3 to 5 independent samples for each strain. Means and standard errors of the means are shown. A Kruskal-Wallis test was used to analyze the data (*, p value < 0.05; ***, p value < 0.001).

The amounts of methyl-ester fatty acids appeared to be somewhat higher in BPSM extracts than in BPRM1 extracts, even though the difference was not significant according to the results of a non-parametric Kruskal-Wallis statistic test. However, BPRM1(pBP2936), which carries *BP2936* on the complementation plasmid, produced significantly higher levels of methyl-ester fatty acids than the other strains (Fig 4). This is consistent with the immunoblot data, showing that 2936P-wt was produced at higher levels than in the parental strain when the deletion was complemented from a plasmid (Fig 2A). This result thus corroborates the trends observed in the comparison between BPSM, BPRM1 and BPRM2 (Fig 4). In contrast, very low levels of methylated fatty acids were detected in the strain producing the mutant protein 2936P^DA/YS^, similar to those found in the BPRM1 strain, even though the mutant protein was overproduced in the former (Fig 4). These data thus support the hypothesis that *BP2936* codes for a methyl-transferase that esterifies fatty acids in *B*. *pertussis*. We propose to name this gene *fmtB*, for fatty acid methyl-transferase of *Bordetella*. We cannot formally rule out the possibility that *fmtB* has an indirect effect on the amounts of methyl-ester fatty acids in *B*. *pertussis*, but the X-ray and mutagenesis data make this alternative explanation less likely.

### *BP2936/fmtB* in host-pathogen interactions

**Fig 5:**
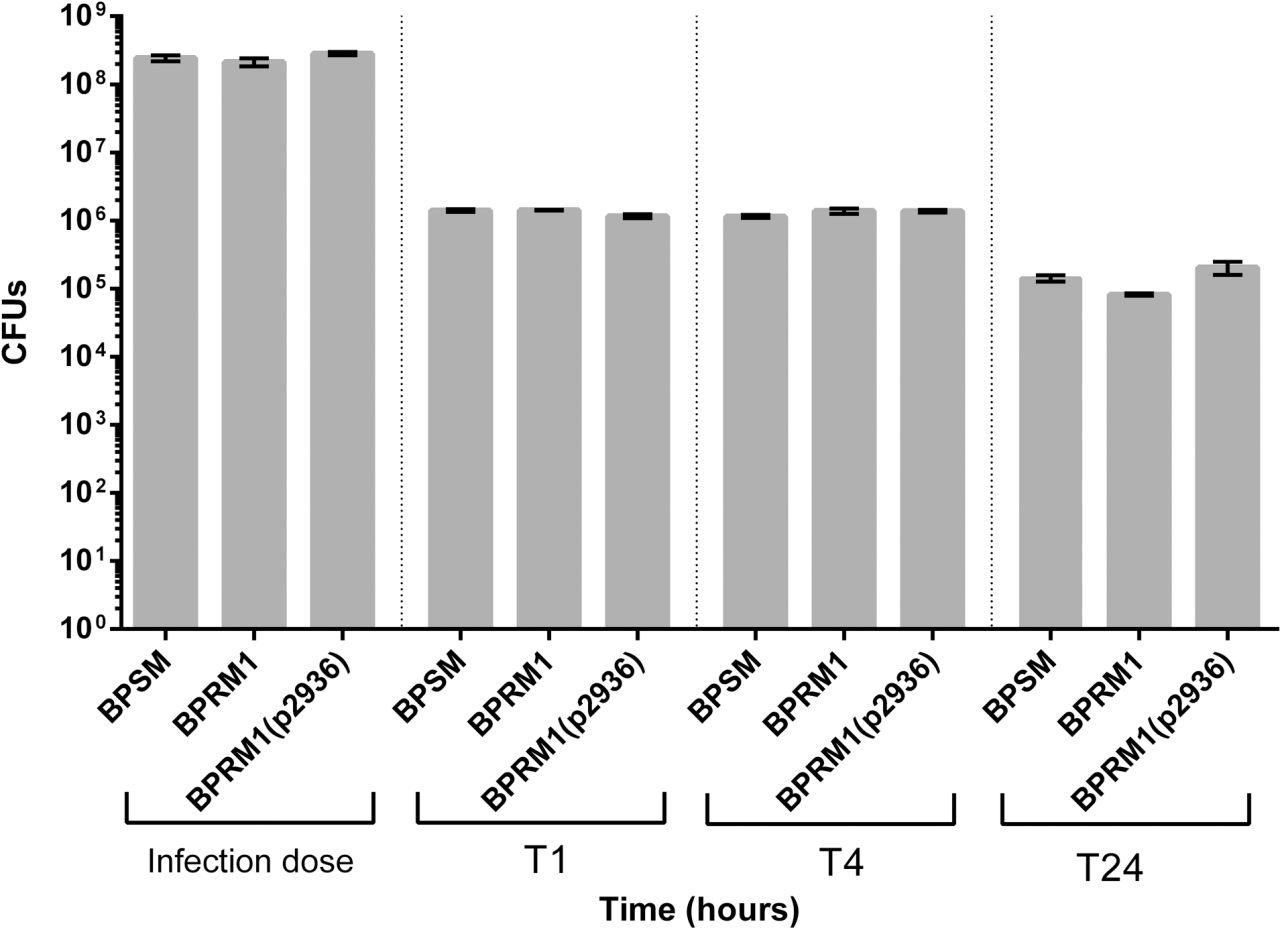
Intracellular survival of *B*. *pertussis* in THP1 macrophages. The BPSM, BPRM1 and BPRM1(pBP2936) strains were used in this experiment. The colony forming units were determined at each time point, expressed in hours after bacteria-macrophages contacts. The experiments were performed in triplicates, and the means and errors of the means are indicated.

Interestingly, methyl-palmitate was reported to inhibit phagocytosis [40–42]. As *B*. *pertussis* is a mainly extracellular pathogen that poorly survives internalization by phagocytic cells [43,44], we reasoned that the FmtB enzyme might interfere with internalization of the bacteria by macrophages. We thus determined the numbers of bacteria internalized upon contact with human THP1 macrophages, and their survival rates over the first hours. BPSM, BPRM1 and BPRM1(pBP2936) bacteria were incubated with THP1-derived macrophages. After killing the extracellular bacteria, the viable bacteria inside the macrophages were counted, and their survival was followed over a few hours time. Very few bacteria survived the internalization, and no significant difference was observed between the three strains (Fig 5).

**Fig 6:**
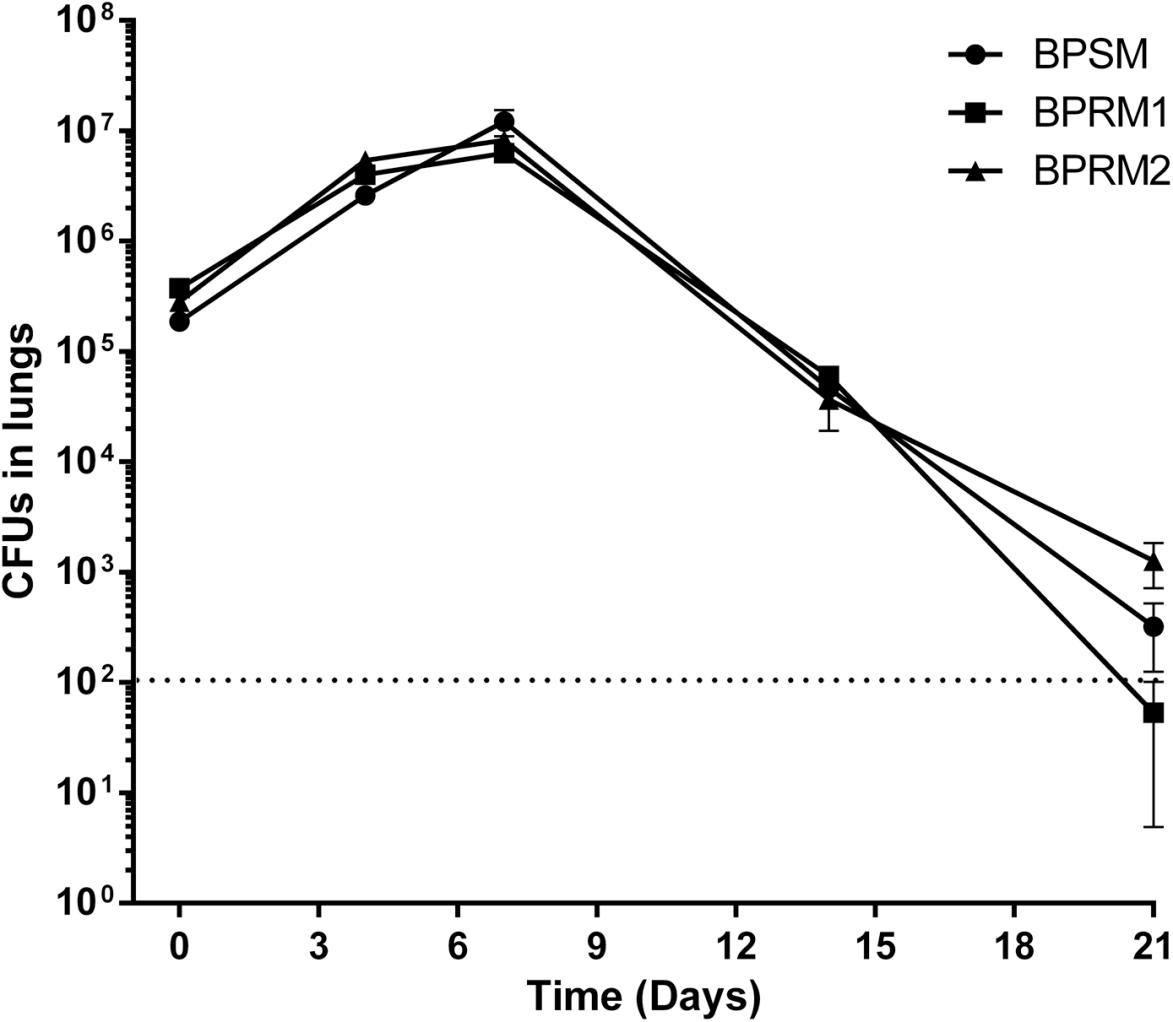
Effect of *BP2936* deletion on colonisation of mice lungs by *B*. *pertussis*. BPSM, BPRM1 and BPRM2 were used to inoculate BalB/c mice intranasally. The mice were sacrificed at the indicated time points (in days), and the viable bacteria present in the lungs were counted. The dashed line indicates the lower limit for the counts of bacteria in the lungs. The means and errors of the means are indicated.

Animal infection experiments were also performed. Balb/c mice were infected intranasally with 10^6^ BPSM, BPRM1 or BPRM2 bacteria, and the numbers of bacteria present in the lungs of the animals were determined after 3 h and 4, 7, 14 and 21 days. Bacterial multiplication occurred after one week, followed by a progressive clearance. This typical colonization kinetics did however not differ between the three strains tested (Fig 6).

**Fig 7:**
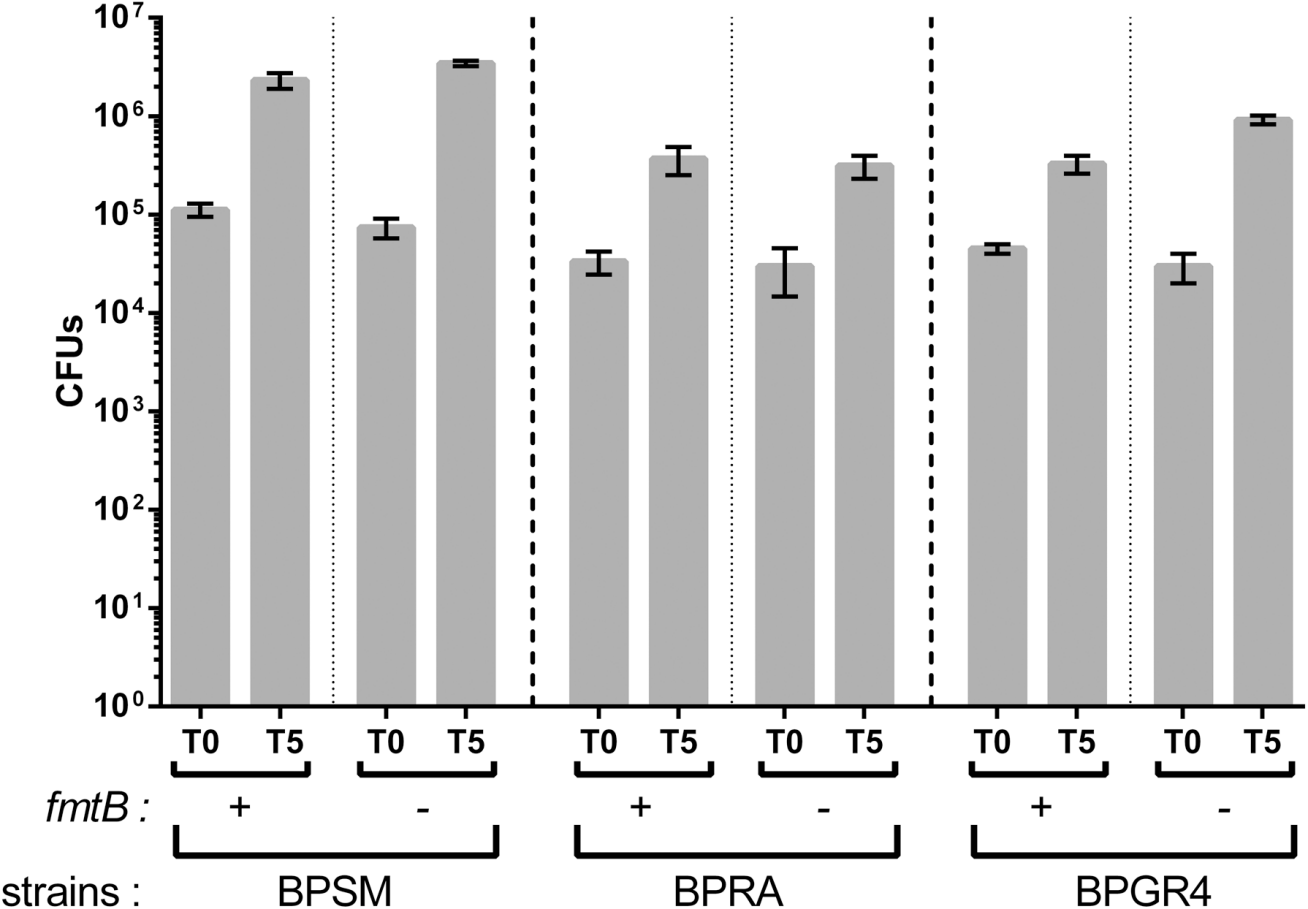
Colonisation of mice lungs by mutant *B*. *pertussis* strains. BPSM, BPRA (*ptx* KO), BPGR4 (*fhaB* KO) strains and their respective derivatives in which *BP2936* (*fmtB*) was deleted, were used in this experiment. Counts of viable bacteria found in the lungs were performed 3 hours (T0) and 5 days (T5) after infection. The means and errors of the means are indicated.

It has been reported that all the virulence factors are not necessary for mouse colonization by *B*. *pertussis*, and that the role of some of them might be masked by the presence of others even if they do not play similar roles in the infection [45]. We reasoned that by weakening the pathogenic potential of the bacterium a putative minor contribution of FmtB to pathogenesis might be revealed. Thus, we tested whether the effect of FmtB could be uncovered in the absence of either of two major virulence factors of *B*. *pertussis*, filamentous hemagglutinin or pertussis toxin. We inactivated *fmtB* by allelic exchange in BPGR4 (*fhaB* KO) and BPRA (*ptx* KO). Animal colonization experiments were performed with the resulting mutant strains, using BPGR4 and BPRA as controls. As reported previously, bacterial multiplication was more limited for the strains that do not produce pertussis toxin or FHA [45]. Nevertheless, the mutants harboring the *fmtB* deletion behaved similarly as their respective parental strains (Fig 7). Altogether, thus, the *in vivo* and *ex vivo* experiments have not revealed a role for FmtB in host-pathogen interactions thus far, at least with the models used in this study.

## Discussion

Among new members of the Bvg regulon revealed by transcriptomic analyses, *BP2936*, which we propose to name *fmtB*, appears to be present in the overwhelming majority of the sequenced strains of the *bronchiseptica* complex. It is also found in other pathogenic *Bordetella* species, but not in non-pathogenic *B*. *petrii*. We showed here that the protein coded by this gene is produced under laboratory growth conditions. This is consistent with the results of two distinct proteomics experiments [33,46].

The *B*. *parapertussis* ortholog of FmtB protein has a typical fold of SAM-dependent methyl-transferases. The identification of a palmitic acid in its enzymatic cavity, the high level of identity between FmtB and its ortholog and results presented in this study strongly support the hypothesis that FmtB generates methyl fatty acids in *B*. *pertussis*. We cannot exclude the possibility that its mode of action is indirect, but the available data do not favor this interpretation. Fatty acids are known to be present in free form in *B*. *pertussis* [47,48], and they might thus represent substrates for FmtB.

Nevertheless, our attempts to detect the methyl-transferase activity of the purified protein *in vitro* were unsuccessful. It is unlikely that the recombinant protein produced in *E*. *coli* was misfolded, since its closely related ortholog from *B*. *parapertussis* expressed in the same host crystallized in a perfectly folded form. Substrate presentation in an assay performed in solution might however be problematic, as insoluble fatty acids are likely not available in free form to the enzyme. In this regard, it is noteworthy that in *B*. *pertussis*, most FmtB was found associated with the membrane fraction, and only a small proportion of the protein was present in the soluble fraction. This observation is in agreement with previously reported proteomic data that detected the product of *BP2936* in membrane-enriched fractions of four different *B*. *pertussis* isolates [33]. However, it contrasts with the situation in *E*. *coli*, where the recombinant protein was largely soluble. These observations may indicate that in its natural host, FmtB is recruited to the membrane via non-covalent interactions with another protein or with specific phospholipids, as described for other methyl-transferases [49]. Of note, the cytoplasmic membrane of *B*. *pertussis* is known to contain lipids absent from *E*. *coli* membranes [48,50]. The association of FmtB with the membrane might facilitate its productive interaction with its free fatty acid substrates in *B*. *pertussis*, consistent with the high levels of methyl fatty acids detected in the strain over-producing FmtB. It is also conceivable that FmtB is modified in *B*. *pertussis*, and that this modification affects its localization and its function.

Methyl-palmitate was reported to inhibit phagocytosis [40–42]. As a mostly extracellular pathogen, *B*. *pertussis* does not survive well internalization by phagocytic cells [43,44]. We thus hypothesized that the production of methyl fatty acids by FmtB might help bacteria avoid clearance by phagocytic cells, thus maximizing the number of bacteria for colonization of the respiratory tract. If this hypothesis was correct, one would have expected lower numbers of wt bacteria internalized upon contact with macrophages compared with bacteria without *ftmB*. However, we observed no marked difference at that time point or at other time points in those experiments. It is therefore difficult to draw a conclusion regarding a potential role for FtmB-methylated fatty acids in the interaction between *B*. *pertussis* and phagocytic cells.

The *fmtB* gene is co-regulated with the virulence genes of *B*. *pertussis*, indicating that it may be involved in the pathogenic potential of the bacterium or contribute to its fitness within the host. Nevertheless, our animal colonization experiments did not demonstrate an effect of the *fmtB* deletion, even in the absence of *fhaB* or *ptx*. The loss of a major virulence factor might enhance the role of another factor [45,51], but this was not observed in the case of the *fmtB* deletion. However, mouse colonization is not the ideal animal model, as it does not fully mimic human infection, and therefore a minor effect might be missed. Experiments with better models are needed to assess the role of this new member of the Bvg regulon. It should however be noted that not all genes positively regulated by BvgAS are *bona fide* virulence factors. In fact, the functions of many of them remain to be established. In any case, the strong sequence conservation of *BP2936* suggests that it is involved in the current lifestyle of *B*. *pertussis* rather than being an evolutionary remnant bound to decay.

Interestingly, *fmtB* gene homologs have been identified in plant pathogens such as *R*. *solanacearum* and *D*. *dadantii*. The function of these genes in those organisms remains to be addressed. An ortholog is also found in *L*. *pneumophila*. Notably, the life cycle of this latter bacterium involves two hosts, an amoeba and a mammal. It will be interesting to determine at which stage of the infectious cycle of this pathogen a fatty acid methyl-transferase may be beneficial. This might also provide clues on the role of FtmB in *Bordetella*.

## Supporting information

S1 Fig ARRIVE guidelines checklist. (DOCX)
