# Supplementary material for: Characterization of a Bvg-regulated fatty acid methyl-transferase in Bordetella pertussis
Source: PLoS One. 2017 May 11;12(5):e0176396. doi: 10.1371/journal.pone.0176396 (PMC5426589; doi:10.1371/journal.pone.0176396)
Supplement: S1 Fig — (DOCX) [file pone.0176396.s001.docx]

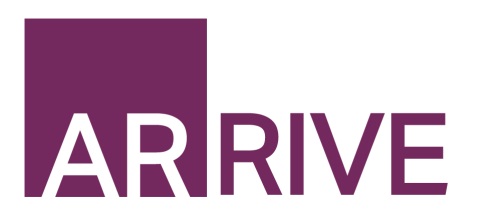


The ARRIVE Guidelines Checklist

Animal Research: Reporting In Vivo Experiments

Carol Kilkenny^1^, William J Browne^2^, Innes C Cuthill^3^, Michael Emerson^4^ and Douglas G Altman^5^

*^1^The National Centre for the Replacement, Refinement and Reduction of Animals in Research, London, UK, ^2^School of Veterinary Science, University of Bristol, Bristol, UK, ^3^School of Biological Sciences, University of Bristol, Bristol, UK, ^4^National Heart and Lung Institute, Imperial College London, UK, ^5^Centre for Statistics in Medicine, University of Oxford, Oxford, UK.*

|  | ITEM | RECOMMENDATION |  |
| --- | --- | --- | --- |


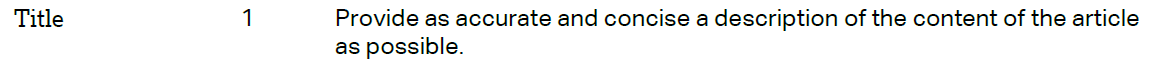


**See title**


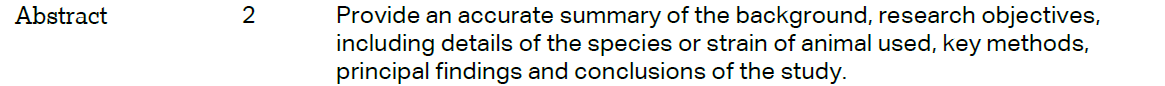


**See abstract**


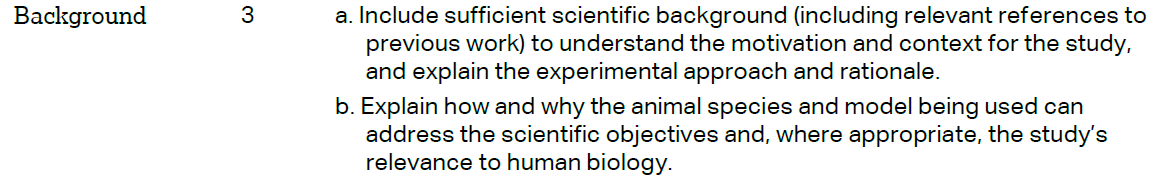


**See introduction; background information is also provided throughout the article (in the results and discussion sections)**


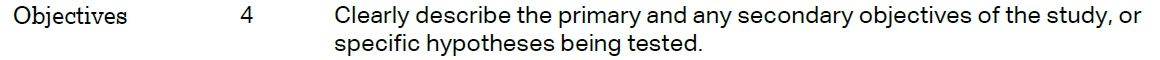


**See paragraph 4 of introduction section.**


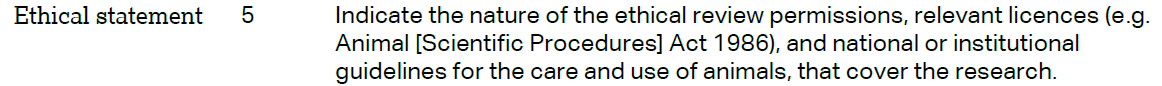


**See last paragraph of the Materials and Methods section.**


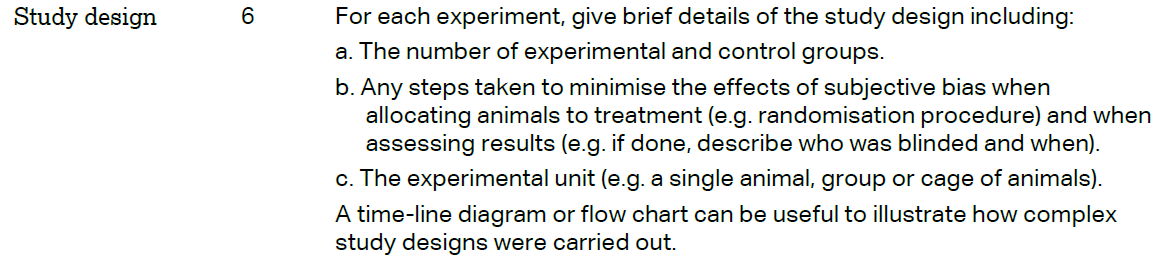
 **In the first experiment, 45 female mice were divided in 3 groups of 15 mice (randomly distributed by the employees of the animal facility at the arrival of the mice). At the time points indicated in line 219, 3 mice per group were sacrificed to determine lung colonization.**

**For the second experiment, 36 female mice were divided in groups of 6 mice (randomly distributed by the employees of the animal facility at the arrival of the mice). At the time points indicated in line 220, 3 mice per group were sacrificed to determine lung colonization.**


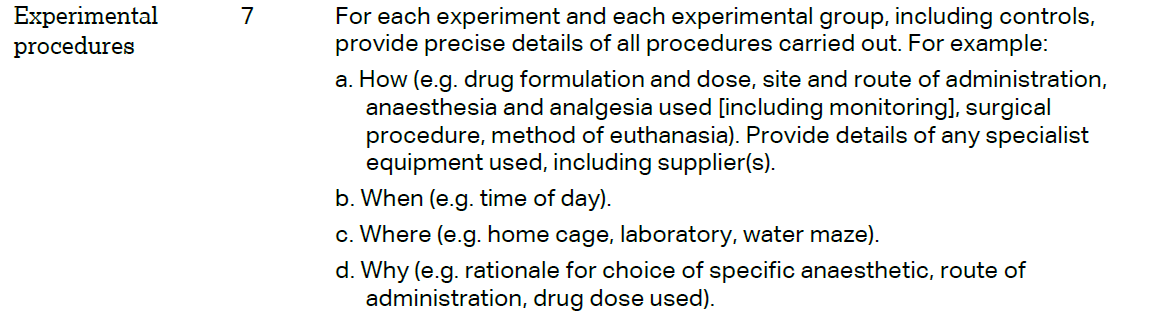
 **Mice were anesthetized intraperitoneally with a mixture of ketamine, atropine and valium (0.03, 0.015 and 0.04675 mg/ml respectively) in the morning, inside a laminar flux hood at the animal biosafety laboratory level 2 (A2) in order to administer the bacterial load by intranasal route, as described in Mielcarek et al. PLOS Pathogens 2006*.**


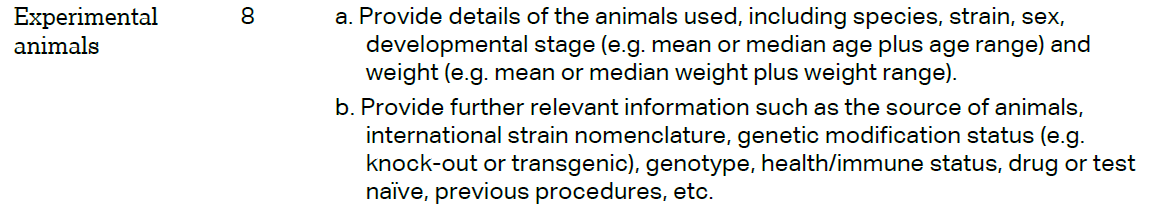
 **6 week-old female JAX^TM^ BALB/cByJ strain of mice were used in these experiments (n=87).**


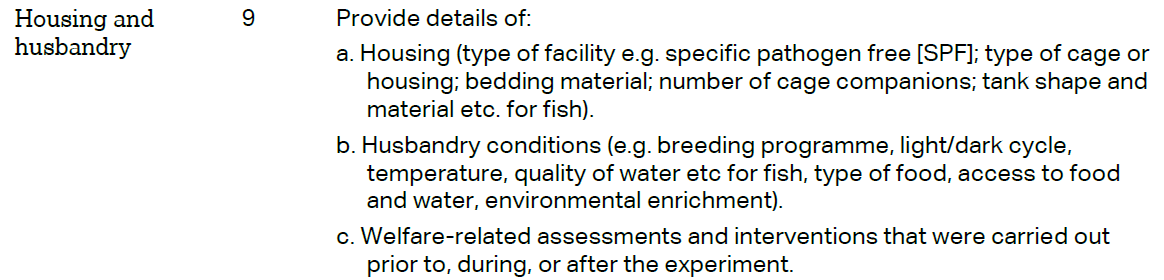
 **Mice were housed at the animal biosafety laboratory level 2 (A2) of the Institut Pasteur de Lille, according to the guidelines of the animal facility. The maximal number of the animals per cage was 5. The Institut Pasteur of Lille has received the agreement for animal experiments from the French Ministry of National Education, of Superior Education and of Research (agreement number B59350009).**


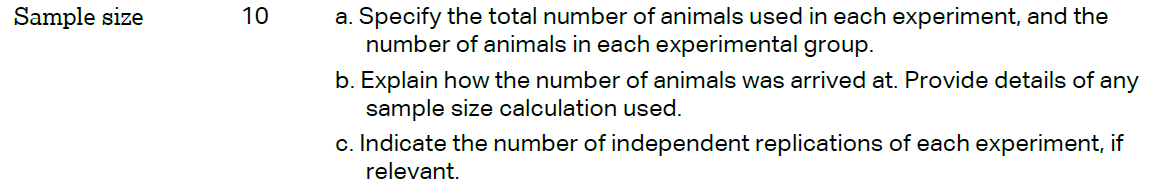
 **In the first experiment, 45 female mice were divided in 3 groups of 15 mice. At the 5 different time points, 3 mice per group were sacrificed to follow the lung colonization.**

**For the second experiment, 42 female mice were divided in groups of 7 mice. At 2 different time points, 3 or 4 mice per group were sacrificed to follow lung colonization.**

**According to previous literature (Mielcarek et al. PLOS Pathogens 2006* and Kammoun et al. Vaccine 2012*) 3 mice per time point are enough to obtain colonization curves with reproducible results.**


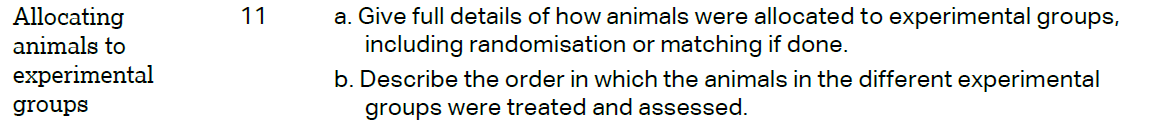
 **Animals were randomly distributed by the people of the animal facility at the arrival of the mice. Mice were infected intranasally with the same numbers of bacteria in 20 μL drops deposited in the nose. On the day of the sacrifice, 3 or 4 mice of each group were randomly euthanized by cervical dislocation.**


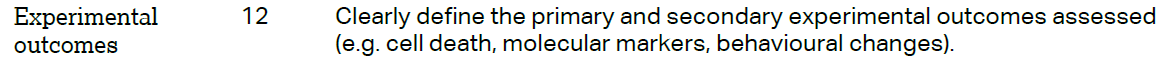
 **Bordetella pertussis does not cause any deleterious effect on wild type mice at the doses of infection used in this work. For this reason, the endpoint for mice was established by the time points selected in the two experiments.**


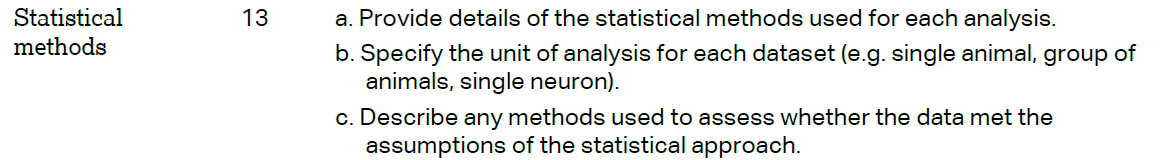
 **All data are reported as the mean ± SEM of three or four measurements.**


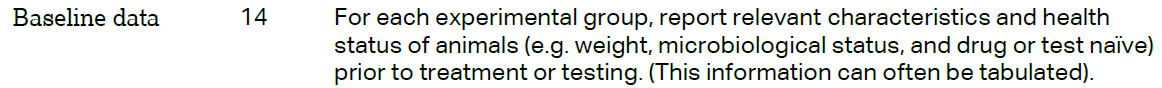
 **During the time period of the different experiments, mice did not presented any appreciable weight loss, behavioral change or prostration.**


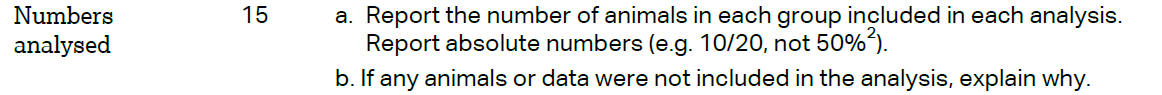
 **Please refer to point #6.**


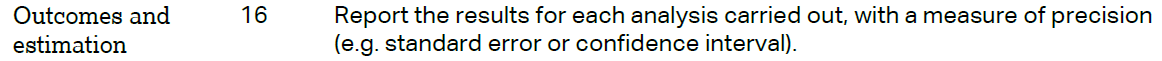
 **All data are reported as the mean ± SEM.**


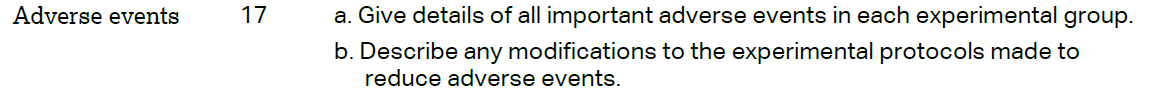
 **No adverse events were observed during the experiments.**


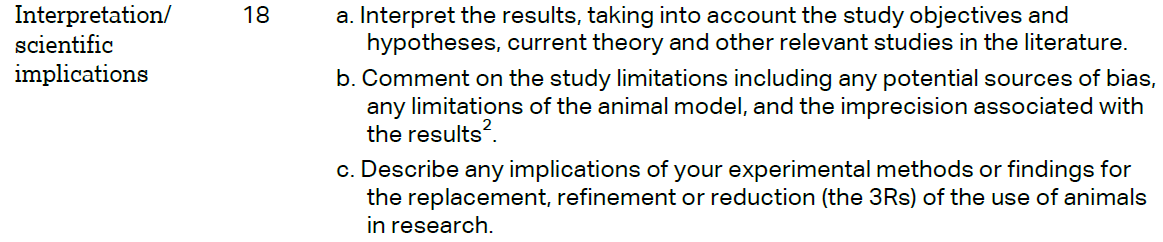


**See paragraph 5 of the discussion section.**


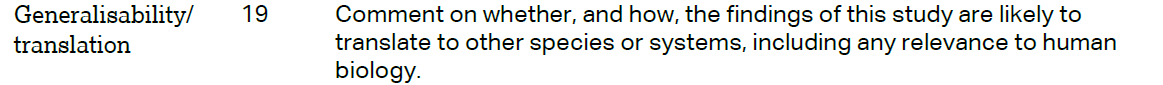


**See last two paragraphs (4 and 5) of the discussion.**


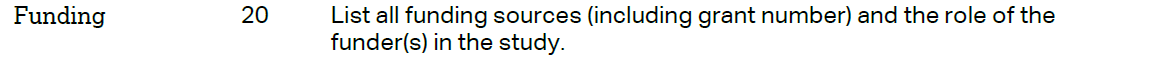


**This work was funded by recurrent funding of our laboratory (INSERM, University of Lille)**

**(see Acknowledgments section)**

**References**

*1.Kammoun, H. *et al.* Dual mechanism of protection by live attenuated Bordetella pertussis BPZE1 against Bordetella bronchiseptica in mice. *Vaccine* **30,** 5864–5870 (2012).

*2.Mielcarek, N. *et al.* Live attenuated B. pertussis as a single-dose nasal vaccine against whooping cough. *PLoS Pathog.* **2,** e65 (2006).
